# Diagnostic comparison between cord blood and filter paper for the screening of congenital hypothyroidism

**DOI:** 10.1002/jcla.24149

**Published:** 2021-12-03

**Authors:** Seham Alameer, Eman Althobaiti, Saud Alshaikh, Meshari Turjoman, Feras Badriq, Abeer AlSofyani, Mohammed Mujalled, Anwar Borai

**Affiliations:** ^1^ Department of Pediatric Ministry of the National Guard‐Health Affairs King Abdullah International Medical Research Center King Saud bin Abdulaziz University for Health Sciences Jeddah Saudi Arabia; ^2^ King Abdullah International Medical Research Center King Saud bin Abdulaziz University for Health Sciences King Abdulaziz Medical City Ministry of National Guard Health Affairs Jeddah Saudi Arabia; ^3^ King Abdullah International Medical Research Center King Saud bin Abdulaziz University for Health Sciences Prince Mohammad ben Abdul‐Aziz Hospital Ministry of National Guard Health Affairs Al‐Madinah Saudi Arabia

**Keywords:** congenital hypothyroidism, cord blood, filter paper, heel prick, TSH

## Abstract

**Background:**

Cord‐blood and heel‐prick TSH levels are essential in diagnosing and preventing the serious complications of congenital hypothyroidism, which mainly include intellectual disability. The study aimed to compare between cord‐blood and heel‐prick TSH sensitivity and specificity in detecting congenital hypothyroidism (CH) among newborn screened babies.

**Method:**

The study included 21,012 newborn screened babies for congenital hypothyroidism starting from September 2013 until March 2019. Both cord‐blood and heel‐prick TSH were collected from each newborn. Heel prick and cord‐blood TSH cutoff values of >21 μU/ml and >30 mIU/L respectively were considered positive.

**Results:**

Out of the total screened newborns, 12 were confirmed for having primary congenital hypothyroidism. Nine cases were positive for cord‐blood TSH (Sensitivity 75%, specificity 99.9%, and a recall rate of 0.004%), while 139 cases were positive for heel‐prick blood TSH (Sensitivity of 100%, specificity of 99.3%, and a recall rate of 0.60%).

**Conclusion:**

For the screening of CH, heel prick is considered a superior method, but cord blood remains a practical option due to its cost‐effectiveness, immediate action, and lower recall rate. Therefore, whenever recall is difficult and/or early discharge is the practice, cord blood is an alternative method to heel prick but not with cases of prematurity.

## Graphical abstract

Cord blood and heel prick TSH levels are essential in diagnosing and preventing congenital hypothyroidism (CH). Out of the total screened newborn 12 were confirmed for having primary congenital hypothyroidism. 10 cases were positive for cord blood TSH (Sensitivity 75%, specificity 99.9%, and a recall rate of 0,004%) while 139 cases were positive for heel prick blood TSH (Sensitivity 100%, specificity 99.3%, and a recall rate of 0.60%). Cord blood is an alternative method to heel‐prick but not with cases of prematurity.

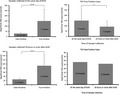

## 1 INTRODUCTION

The human brain requires thyroid hormones for its growth and development, which are essential for intact neurologic functions especially during the first 3 years of life. Therefore, abnormalities in thyroid functions might lead to severe neurological and developmental consequences, one of which is intellectual disability.^1^ To some extent, the most prevalent predisposing factor to intellectual disability is congenital hypothyroidism (CH), which is referred to the deficiency in thyroid hormone since birth. It could be either transient or persistent deficiency. Persistent deficiency of thyroid hormone requires life‐long thyroid hormone replacement. This state is known as permanent congenital hypothyroidism. On the contrary, a temporary deficiency of thyroid hormone which usually improves in a few months is known as transient congenital hypothyroidism.^2^ Prevalence of congenital hypothyroidism in infants with low and very low birth weight is significantly high and is said to be around 1 in 400 cases, but in the case of full‐term infants, it is 1 in 4000 cases.^3^ Almost all infants with congenital hypothyroidism are asymptomatic at birth and show no signs, and thus, the diagnosis is delayed due to a lack of clinical findings in most cases in the newborn period. Eventually, this will prompt the most serious result of congenital hypothyroidism, intellectual disability.^4^ Therefore, screening programs and better management plans have been established to prevent and overcome this disease.^3^

In the 1970s, hypothyroidism neonatal screening programs were developed worldwide.^1^ Cord‐blood specimens and spotted heel‐prick blood on filter papers have been utilized to measure thyroid‐stimulating hormone (TSH) and free thyroxine (FT4) for the screening of congenital hypothyroidism.^1^ The initiation of general screening in the 1970s has effectively reinforced the capacity of North America, Europe, partly Asia, Latin America, and a couple of African nations to surpass congenital hypothyroidism consequences and rise the number of survivors, which helped roughly in diagnosis and treatment of congenital hypothyroidism at an early manner.^1^

In 1972 Dussault, Quebec‐Canada, the first congenital hypothyroidism screening was performed. Seven hypothyroid infants were detected among 47,000 newborns within 3 years. In the initial report, the method missed 10% of the cases with hypothalamic‐pituitary hypothyroidism. It is attributable to under‐developed hypothalamic‐pituitary axis in that group of newborns results in the delayed rise of TSH.^3^, ^4^ Neonatal TSH physiological surge increases TSH levels and prompts dynamic thyroxine (T4) and triiodothyronine (T3) changes within 24 to 48 h from birth. Thus, most centers collect heel‐prick blood samples following 24 h of age to limit the rate of false‐positive high TSH.^4^ In congenital hypothyroidism diagnosis, TSH was more specific. On the contrary, in the detection of hypothalamic‐pituitary hypothyroidism, T4 was more sensitive. Nevertheless, T4 along with TSH is not cost‐effective methods of screening; therefore, generally, TSH and infrequently T4 screening is utilized worldwide.^4^ Hence, optimum sensitivity and specificity of the screening method, cord blood, or heel‐prick blood, is needed especially for high‐risk newborn.^3^

In short, advancements in laboratory research have empowered clinicians to enhance the lives of newborns with congenital hypothyroidism. The development of sensitive and specific assays to measure TSH using cord blood and heel‐prick blood made it possible to initiate highly cost‐effective newborn thyroid screening programs. Thus, early diagnosis and treatment will save detected children from intellectual disability. Therefore, this study aims to compare and determine the sensitivity and specificity of cord blood and heel‐prick blood thyroid‐stimulating hormone (TSH) in detecting congenital hypothyroidism among newborn screened babies at King Abdulaziz Medical City, Jeddah, Kingdom of Saudi Arabia.

## 2 METHODS AND MATERIALS

### 2.1 Data collection

**FIGURE 1:**
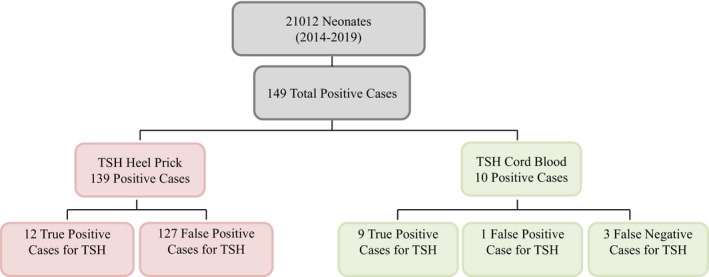
Flow chart showing the total number of neonates included in the study and the number of TSH positive samples collected by both heal prick and cord blood

We conducted a comparative cross‐sectional study at King Abdulaziz Medical City (KAMC), Jeddah, Saudi Arabia. All the data included in the study were obtained from samples, which were delivered from the delivery unit in the hospital. All newborn screened babies from September 2013 until the end of March 2019 were included in this study. We excluded transferred patients from other hospitals, neonatal death prior to specimen collection for newborn screening, babies with incomplete screening for congenital hypothyroidism, and all lost to follow‐up patients. As shown in Figure 1, the population number was 21,012 babies calculated by Raosoft^TM^ software. A non‐probability consecutive sampling technique was used.

The data of patients were collected using a data collection sheet containing the basic demographic variables (gender and date of birth), gestational age, birth weight, initial cord‐blood TSH result, initial heel‐prick TSH results, repeated cord‐blood TSH result, repeated heel‐prick TSH result, confirmed diagnosis, and patient remarks. Heel‐prick TSH blood sample levels of more than 21.0 μU/ml were considered positive and required confirmation. Cord TSH levels above 30.0 mIU/L were considered positive.

The ethical committee at King Abdullah Medical City had granted ethics approval for the study (IRB# SP19‐457‐J). As data would be collected from health records, informed consent was not applicable, and all patients’ medical record numbers were anonymized during data analysis.

### 2.2 Laboratory methods

For cord blood, TSH was performed using i2000 Architect chemiluminescent immunoassay, (Abbott Diagnostics) since 2013 until now. TSH measurement in heel‐prick sample using dry blood spot filter paper was performed using Genetic Screen Processor by Perkin Elmer method.

### 2.3 Statistical analysis

We used proportions for categorical data, means ± SD or median (range) for quantitative data, chi‐square or Fisher exact test for correlating categorical variables, Student's *t* test for correlating qualitative and quantitative variables, and Spearman's correlation for correlating quantitative variables.

## 3 RESULTS

### 3.1 Demographics and characteristics

Our study had a total of 149 patients who had positive heel prick or positive cord blood or both. Furthermore, it stresses that CH is a rare diagnosis with only 12 true‐positive cases. While heel‐prick positive cases were comparable in terms of birth weight (53% normal; 42% low birth weight; 4% very low birth weight; and 1% extremely low birth weight), gestational age (50% term; 44% moderate late/preterm; 5% very preterm; and 1% extremely preterm), and gender distribution with 57% females, cord‐blood‐positive cases were only term (100%) with normal birth weight (100%) and were predominantly females (70%).

### 3.2 True positive vs. false positive

Table 1 depicts TSH levels based on gender, birth weight, and gestational age in true‐positive cases. Results show that male, term babies with normal birth weight had higher TSH levels in both heel prick and cord blood in comparison with others. On the contrary, Table 2 shows that in false‐positive cases, the TSH levels were lower regardless of gender, birth weight, and gestational age. Nevertheless, prematurity and low birth weight increase false‐positive rate in heel‐prick TSH.

**TABLE 1:** True‐positive results for thyroid‐stimulating hormone levels using heel prick and cord blood

Factor	True‐positive results‐TSH heel prick (*N* = 12)		True‐positive results ‐TSH cord blood (*N* = 9)	
	*N*	Mean ± SD	*N*	Mean ± SD
Gender				
Male	3	209.43 ± 156.86	2	552.06 ± 248.90
Female	9	97.76 ± 54.65	7	191.28 ± 152.76
Birth weight				
Normal	9	151.04 ± 97.72	9	271.44 ± 224.82
LBW	1	28.3 ± 0		
VLBW	0			
ELBW	2	60.26 ± 30.85		
Gestational Age				
Term	9	151.04 ± 97.72	9	271.44 ± 224.82
Moderate/late preterm	1	28.3 ± 0		
Very preterm	1	82.08 ± 0		
Extremely preterm	1	38.44 ± 0		

Note

Normal: 2500–4000 grams; LBW: 2500–1500 grams; VLBW: 1500–1000 grams; ELBW: weight less than 1000 grams; term (37 to 42 weeks); moderate to late preterm (32 to 37 weeks); very preterm (28 to 32 weeks); extremely preterm (less than 28 weeks).

**TABLE 2:** False‐positive results for thyroid‐stimulating hormone levels using heel‐prick method

Factor	Heel prick (*N* = 127)	
	*N* (%)	TSH mean ± SD
Gender		
Male	57 (45%)	28.17 ± 7.52
Female	70 (55%)	27.75 ± 6.57
Birth weight		
Normal	64 (50%)	27.65 ± 6.84
LBW	58 (46%)	28.43 ± 7.316
VLBW	5 (4%)	25.91 ± 5.369
ELBW	—	—
Gestational age		
Term	60 (47%)	28.14 ± 8.43
Moderate/late preterm	61 (48%)	27.83 ± 5.58
Very preterm	6 (5%)	26.91 ± 4.06
Extremely preterm	—	—

Note

Normal: 2500–4000 grams; LBW: 2500–1500 grams; VLBW: 1500–1000 grams; ELBW: weight less than 1000 grams; term (37 to 42 weeks); moderate to late preterm (32 to 37 weeks); very preterm (28 to 32 weeks); extremely preterm (less than 28 weeks)

### 3.3 False negative

Table 3 shows three positive CH cases that were detected within cord‐blood sampling as false negative. All three cases were below the average birth weight with preterm in the first and second cases and late preterm in the third case.

**TABLE 3:** False‐negative results for thyroid‐stimulating hormone levels using cord blood

Gender	Birth weight	Gestational age	Heel‐prick TSH	Cord‐blood TSH	Dried blood sample result
			Cutoff < 21 uU/ml	cutoff ≤ 29.99 uU/ml	Cutoff < 21 uU/ml OR plasma/urine value
Female	ELBW	Extremely preterm	38.44	4	Remarkable
Female	ELBW	Very preterm	82.08	3	Remarkable
Male	LBW	late preterm	28.3	10	Remarkable

Note

Normal: 2500–4000 grams; LBW: 2500–1500 grams; VLBW: 1500–1000 grams; ELBW: weight less than 1000 grams; term (37 to 42 weeks); moderate to late preterm (32 to 37 weeks); very preterm (28 to 32 weeks); extremely preterm (less than 28 weeks)

### 3.4 Sensitivity and specificity

As shown in Table 4, heel‐prick samples have 100% sensitivity with higher recall rate and much lower positive predictive value, while cord‐blood samples which have higher specificity (99.9%) but lower sensitivity (75%).

**TABLE 4:** Test efficacy of the cord blood and heel prick for thyroid‐stimulating hormone

	TSH heel prick	TSH cord blood
Sensitivity	100%	75%
Specificity	99.3%	99.9%
Recall rate	0.60%	0.004%
Positive predictive value	8.63%	90.00%
Diagnostic accuracy	99.39%	99.99%

### 3.5 Time of heel‐prick sample collection

While cord‐blood samples are taken immediately after delivering a child, heel‐prick samples have different collection timing depending on each center. Out of the 139‐positive heel‐prick samples, 121 samples (87%) were collected on the same day of birth. In comparison, only 18 samples (13%) were collected 24 h or more after birth.

**FIGURE 2:**
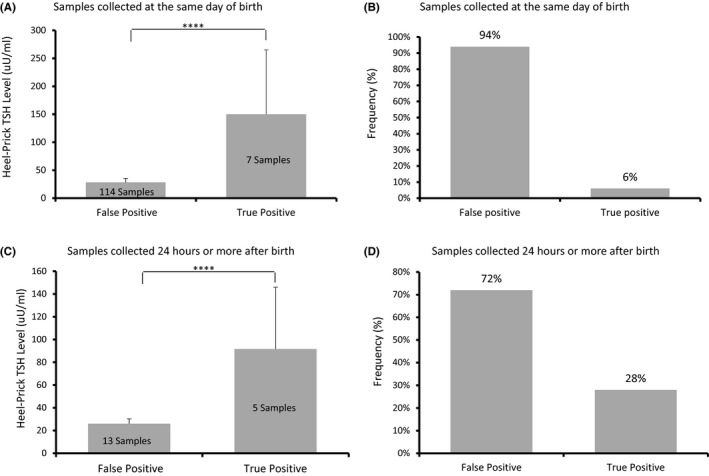
Mean differences of the heel‐prick TSH (uU/ml) between false positive and true positive with their percentages among the samples collected on the same day of birth (A and B) compared with samples collected 24 h or more after birth (C and D)

Moreover, TSH levels in those true‐positive samples were notably higher measuring around 140 uU/ml (Figure 2A). Samples that were collected on the same day had the highest false‐positive rates (94%) with only 7 true‐positive samples (6%) (Figure 2B). TSH level in those true‐positive samples was also less than the TSH level in samples collected on the same day measuring around 90 uU/ml (Figure 2C).

In comparison, samples that were collected after 24 h or more of birth had lower false‐positive rates (72%) with only 5 true‐positive samples (28%) (Figure 2D).

**FIGURE 3:**
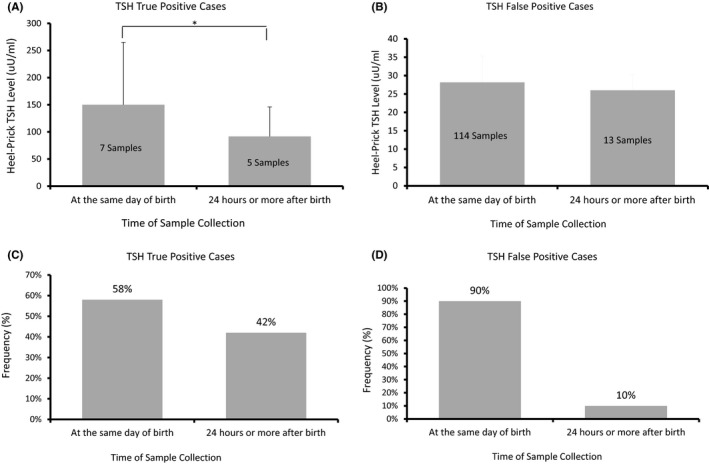
Mean of heel‐prick TSH (uU/ml) in both true‐positive (A) and false‐positive (B) results in relation to the time of sample collection with their percentage of cases in both true positive (C) and false positive (D)

Overall, TSH levels in both heel‐prick samples collected at birthday or 24 h after birth were much higher in true‐positive cases (Figure 3A) compared with false‐positive cases (Figure 3B). True‐positive cases in both heel‐prick samples collected at birthday or 24 h after were comparable (Figure 3C); however, false‐positive cases were much higher when collected on the same day of birth (Figure 3D).

## 4 DISCUSSION

Up to date, this study is one of a few that have compared the sensitivity and specificity between cord and heel‐prick TSH neonatal screening for CH. TSH, which is the main screening test, had been measured through both cord and heel‐prick blood in order to early detect and diagnose CH.

Our study had a total of 149 cases: 10 were detected by cord blood and 139 were detected by heel brick, who had positive heel prick or positive cord blood or both. Furthermore, it stresses that CH is a rare diagnosis with only 12 true‐positive cases. All the cases were true‐positive heel‐brick samples, but only 9 cases were true‐positive cord‐blood samples.

Our data demonstrated that cord TSH results had fewer false‐positive samples compared with heel prick. Al‐Juraibah et al. reported that false‐positive rates were around three times higher in heel‐prick TSH compared with cord blood.^5^ This supports that heel‐prick TSH is inferior to cord TSH regarding positive predictive value. A limitation of Al‐Juraibah et al. study that the effect of birth weight and gestational age was not investigated. However, in our study, birth weight and gestational age were investigated. We found that prematurity and low birth weight comprise half of false‐positive cases in heel prick, which appeared to increase false‐positive rates. Although, term babies with normal birth weight had higher TSH levels in both heel prick and cord blood compared with others. Moreover, in the present study, we found 3 positive CH cases that were detected within cord‐blood sampling as false negative; initially, with low cord‐blood TSH levels, then they were confirmed by repeated TSH sampling. On the contrary, they had high heel‐prick TSH levels. All of them were premature (25 + 6 weeks, 29 weeks, 35 + 6 weeks) and had low birth weight (0.71 kg, 0.99 kg, 1.75 kg), respectively. Similarly, Wong et al. found that out of 22 confirmed CH cases, 5 were premature (28 weeks, 29 weeks, 24 weeks, 34 weeks, 32 weeks) and had low birth weight (0.75 kg, 1.1 kg, 0.595 kg, 1.18 kg, 0.94 kg), respectively, and were detected by second TSH sampling.^6^ Thus, the 3 false‐negative cases in cord blood that were picked up by heel prick are significant because the aim of newborn screening was to detect all affected newborns, which implies having a highly sensitive test at a reasonable specificity.

Mengreli et al. found that many cases of CH were missed at 20 mU\L cutoff. About 40% of them were premature.^7^ Many other studies showed that the relation of neonatal factors and cord‐blood TSH level varies between different populations, and there were recommendations to adjust cutoff values according to the level of prematurity to minimize the high false‐positive rate.

In terms of sensitivity and specificity, 139 neonates had positive heel‐prick TSH samples with 100% sensitivity and 99.3% specificity. Similarly, Al‐Juraibah et al. had 305 positive heel‐prick TSH samples with 100% sensitivity, 98.3% specificity but higher recall rate of 1.68% compared to 0.6% and less positive predictive value of 2.3 compared to 8.63% in our study.^5^ On the contrary, our cord‐blood TSH samples results with 75% sensitivity and 99.9% specificity were compared with the result in Al‐Juraibah et al study with 88 positive samples with 100% sensitivity and 99.6% specificity but similar positive predictive values of 8.63% and 7.95%, respectively, and much lower recall rate.^5^ Though Al‐Juraibah et al. had highly sensitive results, they did not investigate preterm babies who had late TSH rise, which can be simply missed. Therefore, we believe our outcomes are more reliable. In another study, Nasheeda et al. reported 69 positive cord TSH samples with specificity of 94.6% and positive predictive value 7.25%.^8^ A report by Hardy et al. stated that heel‐prick blood TSH has more sensitive and specific than cord TSH with 1 in 1000 recall rate compared to 1 in 23 in sampling with cord TSH.^9^

Our study had a lower recall rate than different studies.^5^, ^10^, ^11^ This was attributed to many reasons such as sample size variation, cutoff values either 20 or 30 mIU/L, TSH analytical methods, and sample collection time. Increasing the cutoff value from 20 to 30 mIU/L made recall rate of many studies drop from 1.83% to 0.91%. In Saudi Arabia, our recall rate of 0.6% intermediated to other studies with recall rates of 0.18% and 1.7%.^5^, ^12^ Cord and heel‐prick TSH sampling are both sensitive but when it comes to low recall rate, cord TSH is more practical than heel prick.

Our study included 21,012 babies who were screened for CH. Out of this number, only 12 babies were confirmed for having CH with an incidence rate of 1:1751. All confirmed cases were positive for heel prick, cord blood, or both. Al‐Juraibah et al. study screened 17,729 babies; 7 cases were confirmed for the diagnosis of CH, which reflects that it is a rare diagnosis to be found.^5^ In term of gender distribution in babies who were suspected for CH (*N* = 139), our study showed a more of female predominance with male to female ratio of (1:3 in TSH Heel Prick and 1:3.5 in TSH cord blood). These are similar outcomes to what was shown in other studies such as Al‐Maghmasi et al. and Henry et al. (1:3 and 1:2 respectively).^13^, ^14^

Our study suggested that the prevalence of CH is comparable to figures such as Bisha province in Saudi Arabia, which showed the highest prevalence in the region, 1:1173 using cord‐blood TSH screening. On the contrary, the province of both Al Baha and Hail represented the lowest prevalence in the country, 1:7709 and 1:6550, respectively.^15^ Our country's CH average prevalence is 1:3293 compared with low prevalence countries such as Kuwait and Japan, which have similar prevalence of 1:7686; however, some countries have higher CH prevalence such as California, USA, and Iran, 1:1706 and 1:748, respectively.^16^, ^17^

## 5 CONCLUSION AND RECOMMENDATIONS

Cord‐blood TSH appears to be more practical option as a screening method for CH disorders due to low cost, low recall rate, and immediate action. Especially in countries where the earliest possible discharge is their current practice, it is challenging to get the newborn back to do the test in the hospital. Healthcare centers that are looking for high sensitivity regardless of the recall rate can use the heel prick as screening method for CH. Heel‐prick test is the superior screening choice for premature/low birth weight babies.

## CONFLICT OF INTEREST

The authors declare no competing interests.

## AUTHOR CONTRIBUTION

SA researched the literature and conceived the idea of the study. EA and AB carried out data retrieval. SA, MT, FB, and MM carried out analytical. AA done statistical analysis. SA and AB finally reviewed the article. All authors participated in reviewing and writing the article.

## Data Availability

The data can be available from the corresponding author upon request.
